# Ferroptosis and its relationship with cancer

**DOI:** 10.3389/fcell.2024.1423869

**Published:** 2025-01-14

**Authors:** Chuanchao Su, Yiwen Xue, Siyu Fan, Xin Sun, Qian Si, Zhen Gu, Jingfei Wang, Runzhi Deng

**Affiliations:** Nanjing Stomatological Hospital, Affiliated Hospital of Medical School, Institute of Stomatology, Nanjing University, Nanjing, China

**Keywords:** PCD, ferroptosis, immune system, tumor progression, tumor treatment

## Abstract

Marked by iron buildup and lipid peroxidation, ferroptosis is a relatively new regulatory cell death (RCD) pathway. Many diseases like cancer, myocardial ischemia-reperfusion injury (MIRI), neurological disorders and acute renal failure (AKI) are corelated with ferroptosis. The main molecular processes of ferroptosis discovered yet will be presented here, along with the approaches in which it interacts with tumour-associated signaling pathways and its uses in systemic therapy, radiation therapy, and immunotherapy managing tumors.

## 1 Introduction

**FIGURE 1:**
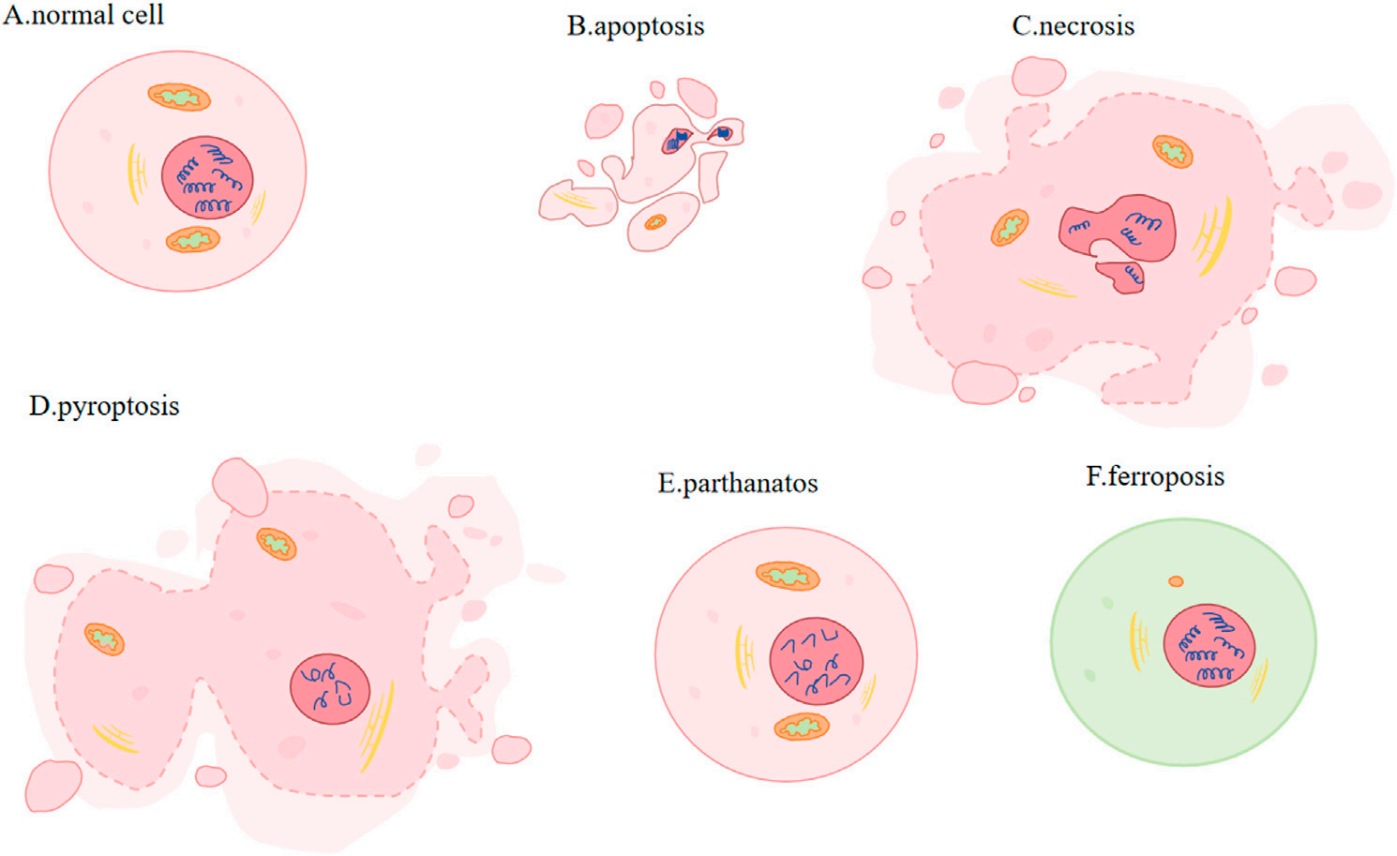
Different types of PCD. **(A)** Normal cell: plasma membrane: complete and stable. **(B)** Apoptosis (activation of caspases): cell and nuclear volume reduction; plasma membrane: blebbing; nucleus: nuclear division and chromatin condensation. **(C)** Necrosis (activation of RIP1/RIP3 and pseudokinase MLKL): increased cell volume, organelle swelling, plasma membrane rupture and subsequent leakage of cell contents. **(D)** Pyroptosis (Caspase1 or Caspase11 mediated gasdermin cleavage): cells expand and form protrusions; plasma membrane: oligomerized and punched; nucleus: nuclear contraction and DNA breakage. **(E)** Parthanatos (large amounts of PAR produced): Nucleus: chromatin condensation and massive production of DNA fragments. **(F)** Ferroptosis (involving Fe^2+^ and lipid peroxidation): Mitochondria: smaller, membrane becomes denser, crista is smaller or disappears.

A strong correlation exists between regulatory cell death (RCD) and the onset, advancement, and management of cancer. RCD is critical for immune response, homeostasis maintenance, embryogenesis and other processes (Allocati N et al., 2015). Distinct from accidental cell death, it is regulated by a variety of signal transduction pathways and is susceptible to genetic and pharmacological interferences, which is (Galluzzi L et al., 2018). Several RCD types, each with their distinct molecular mechanisms, have been found by researchers, including apoptosis, necrotizing apoptosis, pyroptosis, parthanatos and ferroptosis (Tang D et al., 2019) (Figure 1).

The two most common methods for inducing apoptosis are death receptors and mitochondrial pathways. Induced by activation of caspase9 (Fuchs and Steller, 2011) and regulated by Bcl-XL, Bcl-2, Bak, Bax and p53, apoptosis is a process that involves phosphatidylserine shift, volumn reduction, chromatin agglutination and nuclear division. Furthermore, mitochondria will degrade to maintain cellular homeostasis, and excessive ROS will induce autophagy during apoptosis (Guo QQ et al., 2020). Involving activating receptor-interacting protein kinase 1 or 3 (RIP1 or RIP3) and pseudokinase MLKL, necroptotic apoptosis accumulates of ATP, ROS and calcium, changes mitochondria permeability, creats pore, increases cell and organelle volume, ruptures plasma membrane and gets cell content leaked (Vanden Berghe T et al., 2014; Yuan and Kroemer, 2010; Vandenabeele P et al., 2010). In pyroptosis, cells undergo expansion and formation of protrusions. This comes with nuclear contraction and DNA breakage (Bergsbaken T et al., 2009). Its mechanisms of action involves gasdermin cleavage, which releases the N-terminal domain and subequently oligomerizes and perforates the cell membrane (Tang D et al., 2019). Parthanatos is regulated by molecules AIFM1 and PARP1(Poly (ADP-ribose) polymerase-1). Parthanatos is characterized by produceing large amounts of PAR via the activation of PARP1, and it is accompanied by chromatin condensation and the generation of copious amounts of DNA fragments (Wang Y et al., 2009). Caspases, MLKL and GASDM are not involved in ferroptosis (Yang WS et al., 2014). Transmission electron microscopy (TEM) revealed that cells undergoing ferroptosis did not exhibit hallmarks of apoptosis, necrosis, or autophagy. These processes are characterized by chromatin agglutination and marginalization, cell expansion and rupture, and formation of double-membrane closed vesicles. Instead, the mitochondria were observed to be smaller, their membranes were denser and their crista were fewer or absent (Terman and Kurz, 2013; Zhong and Yin, 2015). Its regulatory systems are gradually being discovered over the years and disregulation of ferroptosis has been linked to neurodegeneration, infection, inflammation and tissue damage (Tang D et al., 2021).

## 2 Discovery of ferroptosis and its corralation with various dieseases

In 1980, it was discovered that cystine/glutamate antiporter (system Xc-) which holds great importance for regulating ferroptosis transported cystine into cells by exchanging glutamate out of cells (Bannai and Kitamura, 1980). The key protein solute carrier family 7 member 11(SLC7A11) was also found in the 1980s. In 1997, it was discovered that inhibiting arachidonic acid 12-lipoxygenase (ALOX12), a kind of lipid dioxygenase containing iron, prevented glutamate-induced cell death of hippocampal cell lines HT22 and primary cortical neurons. In contrast, exogenous arachidonic acid (AA), which serves as the substrate of ALOX12, would hasten cell death (Li Y et al., 1997). In 2003, during an experiment involving the screening of tens of thousands of compounds in search of agents that can specifically eliminate tumor cells, a compound called erastin was identified to selectively cause a type of cell demise on tumor cells that are RAS mutant. This specific cell death is non-apoptotic, transpiring rapidly and in an irreversible manner (Dolma S et al., 2003). Researchers identified small molecule 3 and 5 (RSL3 and RSL5) as synthetic complexes that are RAS-selective lethal and could specifically eliminate BJeLR cells in a form other than apoptosis now known as ferroptosis in 2008 (Yang and Stockwell, 2008).

**FIGURE 2:**
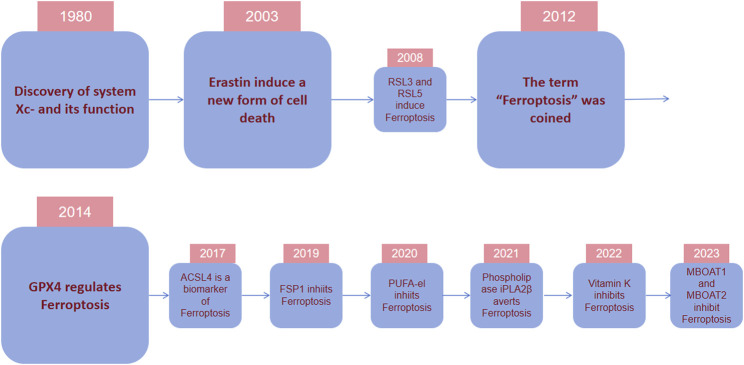
Timeline depicting essential discoveries in ferroptosis research. In 1980, it was discovered that system Xc-, a cystine/glutamate antiporter important for ferroptosis modulation, transports cystine into cells by exchanging glutamate. In 2003, during an experiment screening tens of thousands of compounds for agents to eliminate tumor cells, erastin was identified to selectively cause nonapoptotic, rapid, and irreversible cell death in tumor cells with RAS mutations. In 2012, the term “ferroptosis” was coined because its occurrence depended on the presence of iron. In 2014, it was confirmed that GPX4 regulates ferroptosis. In recent years, various regulatory mechanisms of ferroptosis and their regulatory substances have been gradually discovered.

In 2012, the term for this type of deat was coined as “ferroptosis” because its occurrence hinges on the presence of iron (Dixon et al., 2012) (Figure 2). In the same year, erastin was dicovered to suppress cystine uptake by Xc-, causing ferroptosis. In 2014, Glutathione Peroxidase 4 (GPX4) was confirmed to regulate ferroptosis (Yang WS et al., 2014). A study in 2017 showed that acyl-CoA synthetase long-chain family member 4 (ACSL4) could promote something required for the occurrence of ferroptosis, polyunsaturated fatty acids (PUFAs) (Doll S et al., 2017). The necessity of selenium for GPX4 to inhibit ferroptosis was described in 2018 (Ingold I et al., 2018). In 2019, a novel pathway of ferroptosis inhibition was identified, suggesting that FSP1(ferroptosis inhibitor protein 1), a CoQ10 oxidoreductase, can inhibit ferroptosis and it is independent on glutathione (GSH) (Doll S et al., 2019; Bersuker K et al., 2019). Further investigatios of ferroptosis-sensitive genes through genome-wide CRISPR-Cas9 inhibition screens suggest that oxidative organelle peroxisomes, via using synthetic polyunsaturated ether phospholipids (PUFA-ePL), help cancer cells increase susceptibility towards ferroptosis (Zou Y et al., 2020). In 2022, FSP1-dependent atypical vitamin K cycling was found to curb ferroptosis via inhibiting lipid peroxidation (Mishima E et al., 2022). A study in 2023 identified MBOAT1/2, the phospholipid-modifying enzymes, as ferroptosis inhibitors by employing a genome-wide CRISPR activation screen (Liang D et al., 2023). Subsequent studies on the mechanisms of MBOAT1/2 found that they inhibit ferroptosis via remodeling the phospholipid profile in cells. What’s noteworthy is that they operate without relying on FSP1 or GPX4 (Liang D et al., 2023).

Numerous investigations showed that tumors exhibit different levels of ferroptosis-related genes and proteins, including ACSL4, arachidonate lipoxygenase 15 (ALOX15), ATP synthase, H+ transporting, mitochondrial Fo complex subunit C3, cysteinyl tRNA synthetase, citrate synthase, dipeptidyl-dippeptidase-4, glutaminase 2, lysophosphatidylcholine acyltransferase 3 (LPCAT3), nuclear receptor coactivator 4(NCOA4), ribosomal protein L8, transferrin receptor (TFRC), spermidine/spermine N1-acetyltransferase 1, solute carrier family 1 Member 5(SLC1A5), farnesyl-diphosphate farnesyltransferase 1, CDGSH iron-sulfur domain1, Fanconi anemia complementation group D2, GPX4, heat shock protein beta 1, metallothionein-1G, nuclear factor erythroid 2 like2(NFE2L2), SLC7A11, ER membrane protein complex subunit 2, heat shock protein family A member 5, cyclin-dependent kinase inhibitor 1 in comparison with their corresponding normal tissue (Liu et al., 2017). There are currently drugs in use, such as Temozolomide for glioblastoma and Sorafenib for liver cancer, and there are also clinical trials aiming to target cancer via initiating ferroptosis in tumor (Cheng and Li, 2007).

The etiology and development mechanisms underlying AKI are highly intricate. Researchers have proposed several molecular pathways to trigger or exacerbate AKI. One major mechanism is considered to be ROS-induced kidney injury. Using inducible GPX4-deficient mice in a study found that GPX4 knockout causes AKI, further suggesting the crucial part of ferroptosis plays in AKI (Friedmann Angeli et al., 2014). Martin-Sanchez confirmed the presence of the characteristics of ferroptosis in folate-induced mouse AKI models, which are lipid peroxidation and downregulation of GSH (Martin-Sanchez et al., 2017). Ferrotosis inhibitor ferrostatin-1 inhibited the macrophage infiltration and the downregulation of chemokines such as IL-33, diminished oxidative stress and tubular cell death, along with decreased tissue damage, while the caspase inhibitor had no renal protective effect.

Ferroptosis correlates with MIRI via autophagy-dependent ferroptosis pathway, GPX4, ROS production and endoplasmic reticulum stress (Zhao WK et al., 2021). Ferroptosis inhibitors and iron chelators reduced myocardial fibrosis and infarction size in animal models (Li W et al., 2019). LOX inhibitors are effective in improving cardiac function after MIRI. And it was recently found that Alox15/15-HpETE could aggravate MIRI by promoting ferroptosis of cardiomyocytes (Cai W et al., 2023). Activating SLC7A11 expression in cardiomyocytes can produce “resurrection” (Chen et al., 2017).

It was also found that inhibition of ferroptosis prevented neurodegeneration (Dixon et al., 2012). Researches showed that the occurrence or progress of spinal cord injury, stroke, multiple sclerosis, Parkinson’s disease and Alzheimer’s disease could be affected by ferroptosis (David et al., 2023).

## 3 Occurrence of ferroptosis

Ferroptosis occurs based on a redox reaction called Fenton reaction which involves the basic and biological element Fe. Fenton reaction is a chemical process forming HO∙ from H2O2 via the catalysis of Fe2+, from which Fe2+ turns into Fe3+. As a result, a common oxide in our life, H2O2, turns into a stronger oxide HO∙. And as a free radical, HO∙ would cause positive chain events creating more and more reactive oxygen species (ROS). Fenton reaction was initially applied in the industry work to melt organic waste. Similarly, each cell itself contains loads of H2O2 and Fe ions, so there exists many possibilities of tiny Fenton reactions occurring in cells (Cheng and Li, 2007). Except characterized by iron accumulation, another feature of ferroptosis is that it is driven by lipid peroxidation. ROS, including free radicals (RO∙ and HO∙), peroxides (ROOH and H2O2) and superoxide (O2∙) initiate the oxidation of lipid, especially PUFAs (Dixon et al., 2014). Comparably, free radicals can also create positive chain reactions on lipid peroxidation.

**FIGURE 3:**
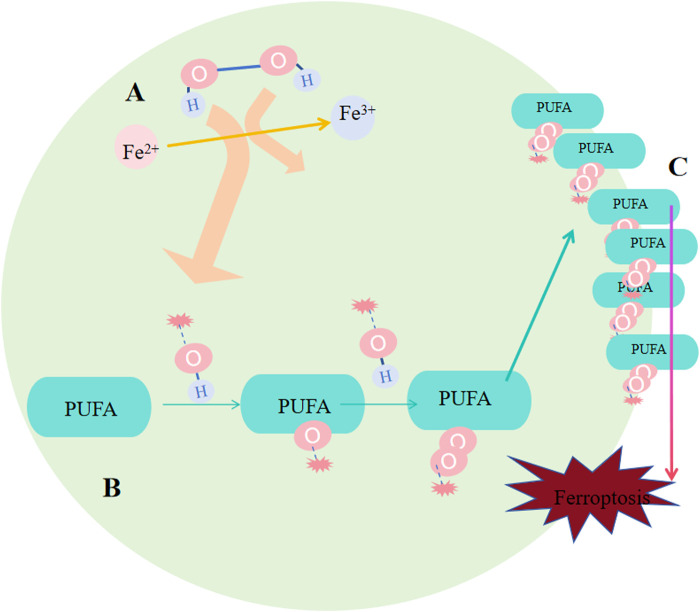
Occurrence of ferroptosis. **(A)** Following the Fenton reaction, hydroxyl radicals (HO∙) were generated from hydrogen peroxide (H2O2) within the cells. **(B)** HO∙ oxidizes lipids to produce RO∙, and RO∙ can be further oxidized into peroxyl radicals (ROO∙). An increasing number of new RO∙ and ROO∙ can be formed based on the preceding HO∙ and RO∙ through the mechanism of positive chain reactions. **(C)** Lipid peroxidation undermines the integrity of the membrane, affecting cell survival because of its crucial role in constituting the biological membrane and maintaining cell shape and function.

Deeep into the details of the process. After Fenton reaction, HO∙ is created from H2O2 in cells. Next, HO∙ will oxidize lipids to produce RO∙, and RO∙ can be further peroxidized into ROO∙ (Figure 3). More and more new RO∙ and ROO∙ then can be generated based on the previous HO∙ and RO∙ via the work of positive chain events (Yin H et al., 2011; Imai and Nakagawa, 2003). The most widely distributed ROS affecting lipids are HO∙ and ROO∙; (Ayala et al., 2014), and they were found to compromise the integrity of lysosomal membrane and thus affect cell survival (Terman and Kurz, 2013). Specific peroxidation of lipid is lethal because of its vital function of composing biological membrane, sustaining cell shape and function. The most important remark of ferroptosis is considerded to be phospholipid hydroperoxide accumulating in cell membrane, but exactly how lipid peroxidation causing ferroptosis needs further investigation. One explanation is that the terminal product of lipid peroxidation, 4-HNE(4-hydroyl-2-nonanal), may induce ferroptosis by creating covalent adducts towards biomacromolecules to wreck the integrity of membrane, leading to proteins crosslink and inactivation, causing membrane rupture (Ayala et al., 2014; Zhong and Yin, 2015).

## 4 Regulation of ferroptosis

### 4.1 Iron metabolism

**FIGURE 4:**
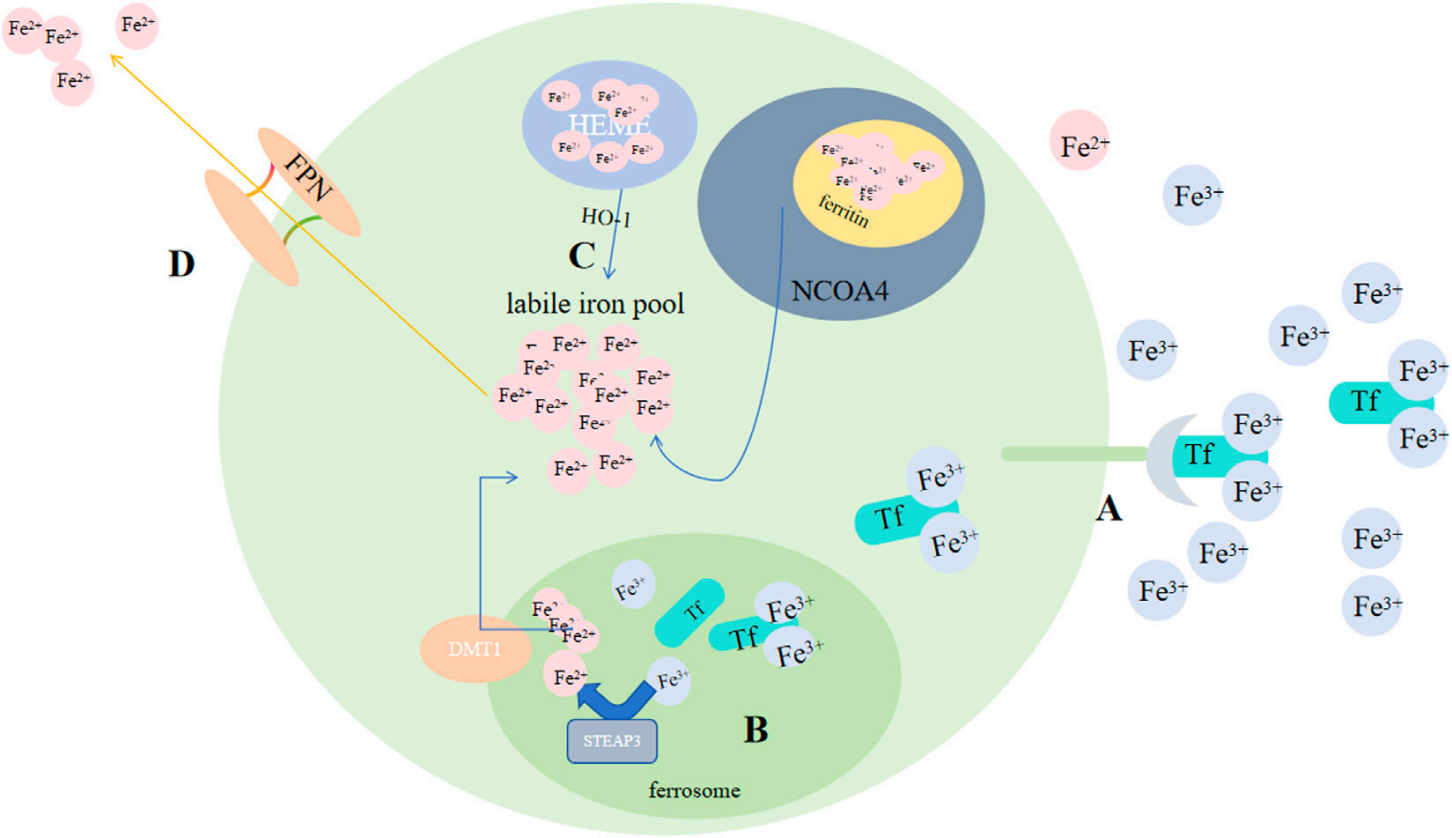
Regulation of ferroptosis by iron metabolism. **(A)** By binding to Tf, Fe^3+^ circulating in the blood can be transported into cells via TFRC-mediated endocytosis. **(B)** After being taken into the cells, STEAP3 on the ferroptosome can reduce Fe^3+^ to Fe^2+^. **(C)** Subsequently, DMT1 could release Fe^2+^ to the LIP, and by binding directly to FTH1; NCOA4 could induce phagocytosis of ferritin, leading to ferritin degradation and an increase in free iron in cells; Catalyzed by HO-1, Fe^2+^ released from heme. **(D)** FPN exports Fe^2+^. Abbreviations: Tf, transferrin; TFRC, transferrin receptor; STEAP3, six-transmembrane epithelial antigen of prostate 3; DMT1, metal-ion transporter 1; LIP, labile iron pool; NACO4, nuclear receptor coactivator 4; FPN, Ferroportin.

Some investigations showed that for organ survival, iron homeostasis playes an essential role: cancer advancemet, drug resistance and metastasis could be promoted by sterol regulatory element-binding protein2(SREBP2) and liver injury, fibrosis and cirrhosis could be suppressed by transferrin (TF) via regulating ferroptosis. In animal models, different interventions on iron metabolism, like limiting iron efflux, decreasing iron storage and enhancing iron absorption, all promote ferroptosis via multiple signaling pathways (Chen X et al., 2020). Iron buildup contributes to lipid peroxidation via 2 methods: activation of Fenton reactions and formation enzymes that contain iron (e.g., lipoxygenase) (Yang WS et al., 2016; Stockwell BR et al., 2017). By binding to TF, Fe3+ that circulate in blood could be imported to cell by TFRC-mediated endocytosis. Thus, serumtransferrin or lactilotransferrin mediated uptake of Fe3+ could promote ferroptosis through TFRC or other receptors (Wang Y et al., 2020; Yang and Stockwell, 2008), while solute carrier family 40 member 1(SLC40A1) could inhibit ferroptosis via exporting iron (Geng N et al., 2018). After being brought into cells, six transmembrane prostate 3 epithelial antigen (STEAP3) on the ferrorosome can reduce Fe3+ to Fe2+(Figure 4). Then, divalent metal ion transporter 1 (DMT1) could release Fe2+ to labile ion pool (LIP) (Yanatori and Kishi, 2019; Zhou B et al., 2020; Muñoz M et al., 2011; Fang X et al., 2020). Ferritin and heme store the majority of iron. FTH1(heavy chain) and FTL(light chain) compose ferritin (Bradley JM et al., 2016). By binding directly to FTH1, NCOA4 could induce phagocytosis of ferritin, causing ferritin degradation and increasing free iron in cells when lacking iron (Dowdle WE et al., 2014; Mancias JD et al., 2014). While exosome-mediated ferritin export could suppress ferroptosis (Brown C W et al., 2019). Catalyzed by HO-1, Fe2+ released from heme would accumulate in cardiomyocytes and initiate ferroptosis (Fang X et al., 2019). BAY and NRF2 may regulate it (Hassannia B et al., 2018; Chang LC et al., 2018). A reduction in hepcidin levels gives rise to tissue iron overload. In response, iron overload, conversely, exerts a positive regulatory impact on the secretion of hepcidin, a peptide hormone generated by liver. Subsequently, hepcidin, in a reciprocal manner, modulates the concentration of Ferroportin1(FPN1) which takes charge of the export of iron on cell surface (Nemeth E et al., 2004). Most cells are unable to export iron efficiently, causing an elevation in the LIP level (Qiu Y et al., 2020).

### 4.2 Enzyme

#### 4.2.1 Cytoplasm: GPX4 and TXNRD1

Two major antioxidant systems exist in most eukaryotes, glutathione system and thioredoxin system. Glutathione system is composed of glutaredoxin (Grx), glutathione reductase (GR) and NADPH; while thioredoxin (Trx), NADPH and thioredoxin reductase (TrxR) compose thioredoxin system. GR or TrxR facilitates the delivery of electrons from NADPH to GSSG or thioredoxin, to a reduced form, thus engaging in several responses for the maintenance of redox balance. It serves as a crucial detoxification mechanism safeguarding cells against reactive oxygen species. GPX4 and TXNRD1 (or Trx1) are two selenoenzymes that are involved in ferroptosis, and they belong to the glutathione system and the thioredoxin system, respectively.

**FIGURE 5:**
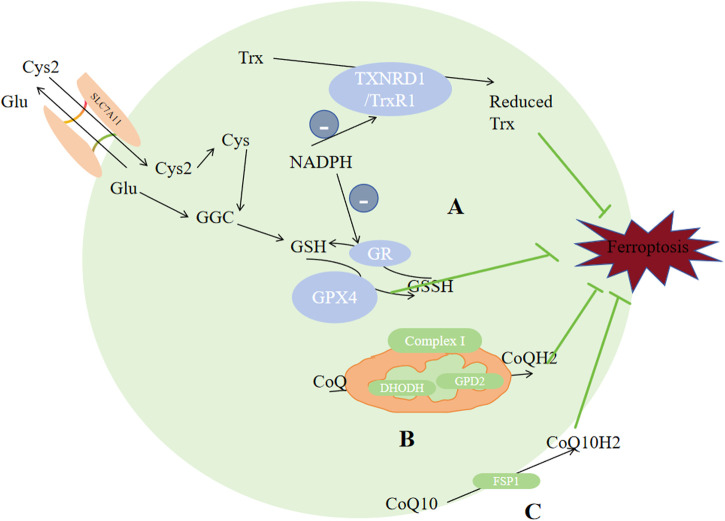
Enzymes regulate ferroptosis. **(A)** GPX4 and TXNRD1 exert antioxidant functions through the metabolism of GSH and reduced Trx. **(B)** CoQ was reduced to CoQH2 by DHODH in the mitochondria. **(C)** CoQ10 was reduced to CQ10H2 by FSP1. Reduced thioredoxin (Trx), glutathione (GSH), CoQH2, and CoQ10H2 are reducing substances capable of compensating for lipid peroxidation which serves as the raw material for ferroptosis. Abbreviations: Cys2, cystine; Cys, cysteine; Glu, Glutamate; SLC3A2,solute carrier family 3 member2; SLC7A11,solute carrier family 7 member 11; GPX4,glutathione peroxidase 4; GR, glutathione reductase; GSH, glutathione; GSS, Glutathione synthetase; GSSH, glutathione counterpart; Trx, thioredoxin; TXNRD1/TrxR1,thioredoxin reductase 1; CoQ10,ubiquinone; FSP1,ferroptosis inhibitor protein 1.

While simultanously reducing harmful phospholipid (PL-OOH) to their corresponding harmless phospholipid alcohol (PL-OH), GPX4 converts GSH to oxidized glutathione (GSSG), thus suppressing ferroptosis (Qiu Y et al., 2020). GPX4 has several function, and it could reduce various forms of peroxidized lipids, including free forms of lipids, phospholipids, lipoproteins and lipids in biological membranes (Brigelius-Flohé and Maiorino, 2013). It is the central antioxidant against ferroptosis because it is the only found to be capable of reducing phosphoperoxides to hydroxyphospholipids in the membane (Wang Y et al., 2020). Ferroptosis would occur when the level of lipid peroxidation surpasses the reducing ability of GSH and GPX4 (Guan J et al., 2009). Synthetic materials of GSH include glutamate, cysteine and glycine (Figure 5). The cysteine content, synthetic activity of glutamate-cysteine complex and feedback inhibition of GSH can all directly regulate GSH synthesis, which would in turn affect the enzymatic activity of GPX4 (Dixon et al., 2012; Hatem E et al., 2017; Bebber CM et al., 2020). Cysteine comes from Xc- and transsulfuration pathway. Xc-consists of the heterodimeric SLC7A11 (xCT) and SLC3A2(4F2hc), exporting glutamate and importing cystine in a 1:1 ratio (Ishii T et al., 1987; Conrad and Sato, 2012; Wang H et al., 2017). It is established that xCT is the crux for ferroptosis regulation (Fang X et al., 2020). NFE2L2 could upregulate xCT expression and activity (Chen D et al., 2017), while tumour suppressor genes like BAP1, BECN1 and TP53 (Song et al., 2018; Jiang L et al., 2015; Zhang Y et al., 2017) could negatively regulate xCT. Once transported into cells, GSH or TXNRD1 could reduce cystine to cysteine (Mandal P. K. et al., 2010). GSH synthesis also requires glutamate, and the absorption of glutamate is dependent on SLC1A5 and SLC38A1 (McGivan and Bungard, 2007). Glutamate-cysteine ligase catalytic subunit (GCLC) catalyzes the initial stage of GSH synthesis via linking cysteine to glutamate (Kang YP et al., 2021). However, GCLC would promote synthetizing γ-glutamyl peptides (γ-Glu-AAs) when cysteine deficiency occurs, which would consume glutamate, inhibiting ferroptosis (Hayano M et al., 2016). Glutathione synthase (GSS) is also involved in the GSH synthesis (Liang C et al., 2019). Through preventing the synthesis of reduced form of GSH, AMPK-regulated beclin-1 phosphorylation enhances ferroptosis (Fang X et al., 2020). In conclusion, factors that regulate ferroptosis, including the biosynthesis and uptake of selenide-l-cysteine (Ingold I et al., 2018; Anderton B et al., 2017) and GSH synthesis (Zhou B et al., 2020; Gao et al., 2015), would simultaneously affect GPX4 homeostasis, backing the notion that GPX4 plays a substantial part in ferroptosis regulation.

TXNRD includes TXNRD1, TXNRD2 and TXNRD3. They are distributed in cytoplasm, mitochondria and testicle respectively. TXNRD1 could reduce Trx, and by using a thiol-disulfide exchange reaction, reduced Trx could further interact with many downstream proteins like enzymes with antioxidant properties and proteins that regulate apoptosis (Balsera and Buchanan, 2019). The TXN system has been demonstrated to exhibit a relatively higher expression level in numerous types of cancers in comparison to that in normal tissues (Zhang J et al., 2017). High dose of AUR (25 mg/kg) could induce ferroptosis via inhibiting TXNRD (Yang et al., 2020a]. Alterperylenol could also induce ferroptosis by inhibiting TXNRD (Xi et al., 2022). So TXNRD inhibitors could be seen as potential targets for tumor management. Other than its direct correlation with ferroptosis, TXN system affects GSH biosynthesis and cystine reduction. If the source of selenium is limited, the bioavailability or utilization of selenium will be distributed between GPX4 and TXNRD1/2, which would regulate the expression of selenoenzymes, and may limit the endogenous synthesis of GPX4, inhibting the ability of reducing lipid peroxidation, since GPX4 plays the central role in inhiting ferroptosis.

#### 4.2.2 Cell membrane: CoQ10 to CoQ10H2

Ubiquinone (CoQ10) is a metabolite of mevalonate, it can be reduced to lipophilic radical scavenger panthenol (CoQ10H2), a common reducing substance in living organism which can compensate for lipid peroxidation. Recent studies identified two antioxidant system against ferroptosis based on this. Through N-terminal myristoylation, FSP1-CoQ10, which act exclusively on GPX4-depleted cells (Zhao L et al., 2022), could be recruited to the plasma membrane, reducing CoQ10 into CoQ10H2 (Shimada K et al., 2016; Bersuker K et al., 2019); Another GPX4-independent system involves GTP cyclohydrolase 1 (GCH1), which would rate-limit BH4(tetrahydrobiopterin) production. BH4 can promote CoQ10H2 formation and block specific lipids peroxidation to inhibit ferroptosis (Mao C et al., 2021).

Except CoQ10, other metabolites of mevalonate could also affect ferroptosis. For example, GPX4 and CoQ10 production require IPP(isopentenyl pyrophosphate). But another product of mevalonate, squalene synthase, can induce ferroptosis through accumulating ROS (Garcia-Bermudez et al., 2019). Derived from statins and CIL56(FIN56), specific ferroptosis inducers can promote ferroptosis via inhibiting the CoQ10H2 and mevalonate pathways to enhance ferroptosis (Shimada K et al., 2016).

#### 4.2.3 Mitochondria: CoQ to CoQH2

ROS in cells mostly come from mitochondrial metabolism. The characteristic morphological alterations specific to ferroptosis are predominantly manifested in mitochondria, including smaller or ruptured mitochondria, dwingdling or vanishing of mitochondrial ridges, and denser mitochondrial membrane. So it is certain that mitochondria plays a significant part in ferroptosis regulation. Interferrences on the TCA cycle, electron transfer chain, and voltage-dependent anion channel 2/3 of mitochondria, could change lipid peroxides buildup level, thus affecting ferroptosis (Yang and Stockwell, 2008; Gao et al., 2019). For example, breakdown of glutamine provides α-ketoglutaric acid (α-KG) to mitochondrial tricarboxylic acid cycle, thereby promoting ferroptosis (Gao and Jiang, 2018; Gao et al., 2015), and depletion of glutamine can weaken the ferroptosis-inducing ability of erastin (Gao et al., 2015). AOA, the transaminase inhibitor, could inhibit ferroptosis via suppressing glutaminolysis. Membrane electron transport proteins, especially POR and NOXs, can increase ROS levels (Tian T et al., 2017; Alim I et al., 2019; Ingold I et al., 2018), promoting ferroptsis. When cysteine insufficiency occurs, mitochondria promotes the quick consumption of glutathione and subsequent lipid ROS generation, thus ferroptosis (Gao et al., 2019).

Some studies proposed a third protective system: DHODH. Distinguished from the former two antioxidant systems which exist in cytoplasma and cell membrane respectively, this enzyme is distributed on the mitochondrial inner membrane’s outer surface, oxidizing DHO(dihydrolactic acid) to OA (lactic acid) and more importanly, reduces ubiquinone (CoQ) to CoQH2, thus inhibiting ferroptosis (Garcia-Bermudez et al., 2019; Mao C et al., 2021). Glycerol-3-phosphate (G3P) dehydrogenase 2 (GPD2) could also reduce CoQ to CoQH2. When CoQ is exhausted by FIN56, the impact of GPD2 knockout on ferroptosis would be weaken (Wu et al., 2022). Complex I, also called NADH dehdrogenase, pumps protons into the intermembrane space, and simultanously transfers electrons to CoQ. Complex II transfers protons from succinic acid to CoQ to generate CoQH2, thus inhibting ferroptosis.

### 4.3 Lipid metabolism

**FIGURE 6:**
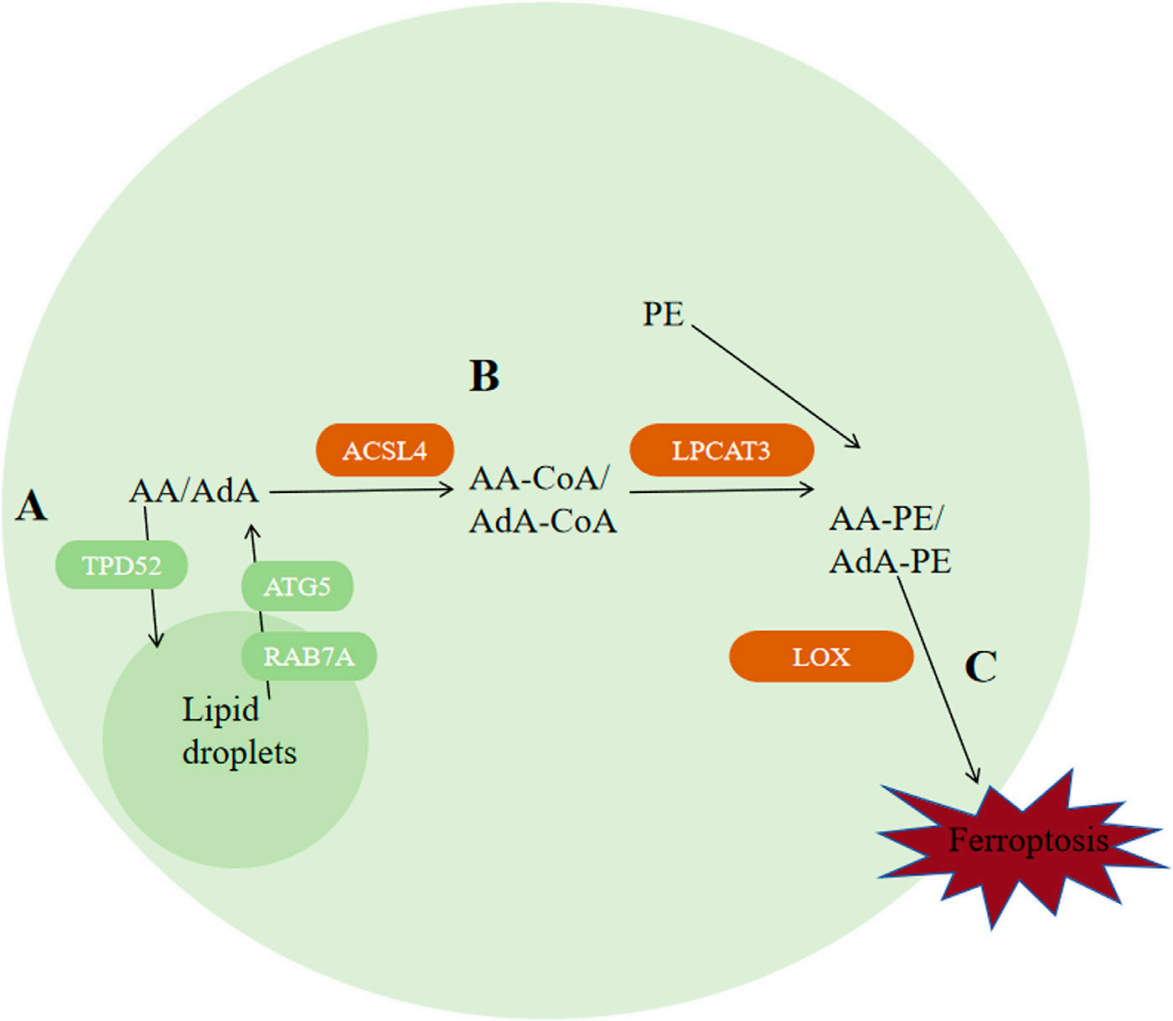
Regulation of ferroptosis by lipid metabolism. **(A)** Autophagy of lipid droplets releases PUFAs, among which AA and AdA are the two types of polyunsaturated fatty acids that are most susceptible to peroxidation. **(B)** ACSL4 catalyzes the binding of free AA and AdA to CoA, generating AA-CoA and AdA-CoA, respectively. Then, LPCAT3 promotes their esterification to react with membrane phosphatidylethanolamine (PE) by specifically binding acyl groups to PE-CoA and phosphatidylcholine (PC), forming AA-PE and AdA-PE. **(C)** PUFAs can be oxidized into their corresponding hydroperoxides by lipoxygenases (LOXs) to produce AA-PE-OOH and AdA-PE-OOH. Abbreviations: ATG5, autophagy-related protein 5; RAB7A, RAS-related protein 7A; AA, arachidonic acid; AdA, adrenal acid; TPD-52, tumor protein D52; ACSL4, acyl-CoA synthetase long-chain family member 4; PE, phosphatidylethanolamine; LOX, lipoxygenase.

Ferroptosis can be inhibited via decreasing autophagy-related protein 5 (ATG5) or RAS-associated protein 7A (RAB7A)-dependent lipid degradation and increasing Tumor protein D52 (TPD52)-dependent lipid storage (Bai Y et al., 2019), indicating that lipid metabolism and ferroptosis are correlated closely. adrenal acid (AdA) and Arachidonic acid (AA) are the two kinds of PUFAs that are most vulnerable to peroxidation. ACAC phosphorylation regulated by AMPK can inhibit ferroptosis by inhibiting the synthesis of PUFAs, while peroxisome-regulated acetal polymerase can promote ferroptosis by providing sources of PUFAs (Figure 6) (Zhang Y et al., 2018; Jiang L et al., 2015; Chen D et al., 2017). Apart from that, enzymes ACSL4, LPCAT3 and arachidonic acid lipoxygenase (ALOX) are engaged during biosynthesis and remodeling of PUFAs in biofilms. ACSL4 facilitates the combination of free AA and free AdA to CoA, generating AA-CoA and AdA-CoA, respectively (Dixon et al., 2012; Bebber CM et al., 2020; Hatem E et al., 2017). ACSL4 expression is regulated by Hippo signaling pathway, radiation therapy, and the lactate microenvironment (Lei G et al., 2020; Wu et al., 2019; Zhao Y et al., 2020). ACSL3 could convert monounsaturated fatty acids towards the corresponding acyl-CoA esters to bind the phospholipids of membrane to inhibit ferroptosis on tumor cells (Magtanong L et al., 2019). Mainly expressed in metabolic tissues like pancreas, fat and liver and distributed in the endoplasmic reticulum (Zhao Y et al., 2008), LPCAT3 promotes their esterification to react with membrane phosphatidyleethanolamine (PE), by specifically binding acyl groups to PE-CoA and phosphatidylcholine (PC), forming AA-PE and AdA-PE (Hashidate-Yoshida et al., 2015). Loss of LPCAT3 greatly induced AA levels in cell membrane and prevented the buildup of cytoplasmic lipid droplets, causing intestinal cell damage and lethality to mice. Last but most importantly, PUFAs can be oxidized into their corresponding hydroperoxides by lipoxygenases (LOXs), producing AA-PE-OOH and AdA-PE-OOH. Different cells requires diferrent lipoxygenases for ferroptosis to occur. Cell lines PANC1, HT-1080 and BJeLR requires ALOX15B, ALOX15, ALOXE3 and ALOX5 (Kang YP et al., 2021; Song et al., 2018; McGivan and Bungard, 2007), while cell line H1299 requires ALOX15 and ALOX12 (Hayano M et al., 2016).

## 5 Tumor ferroptosis and its microenvironment

Complex information exchange networks exist between various type of tumor cells and their surrounding cells. Based on loads of studies, we now know that through immune checkpoint receptors (e.g., PD-1), TNF family death receptors (e.g., TRAIL) and perforin (Gao Y et al., 2017), conventional innate lymphocytes and NK cells can interact with cancer cells. We have found cancer associated fibroblasts (CAFs) can help gastric cancer fight against ferroptosis via secreting miR-522 (Zhang et al., 2020).

### 5.1 Effects of T Cells on ferroptosis of tumor cells

**FIGURE 7:**
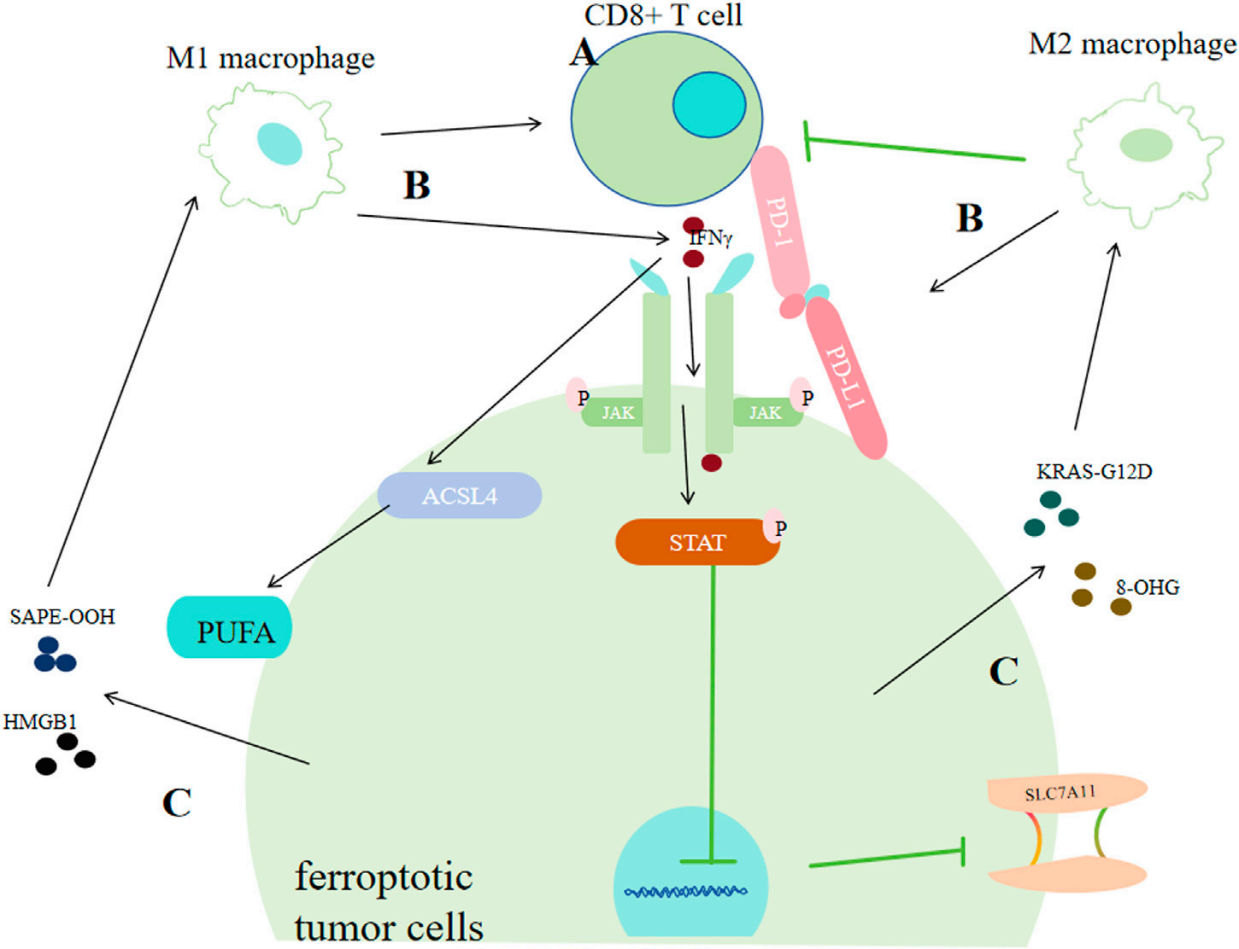
Correlation between tumor cell ferroptosis and the tumor immune system. **(A)** CD8^+^ T cells promote ferroptosis in cancer cells by secreting interferon-γ (IFNγ). IFNγ can strengthen the binding of the xCT transcription start site with STAT1, thus inhibiting its transcription or it can promote ACSL4. **(B)** Macrophages exert dual effects on cancer cells via ferroptosis. M1 macrophages promote ferroptosis by facilitating CD8^+^ T cells or by secreting IFN-γ. M2 macrophages inhibit ferroptosis of cancer cells by enhancing PD-L1 or restraining CD8^+^ T cells. **(C)** Ferroptotic cancer cells exert dual effects on the immune system by releasing DAMPs. Cancer cells can promote the transformation of macrophages into the M1 type by secreting SAPE-OOH or HMGB1, or promote the transformation of macrophages into the M2 type by secreting KRAS-G121D or 8-OHG.

Programmed death receptor 1(PD-1) and programmed cell death 1 ligand 1(PD-L1) is the most extensively known relation between tumor cells and T cells. Tumor cells can show high-level PD-L1 to bind with PD-1 of T cells, causing programmed T cell deaths (Khalil DN et al., 2016; Jiang P et al., 2018), helping themseves adapt to challenges from T cells (Zou et al., 2016; Khalil DN et al., 2016; Jiang P et al., 2018). As for the relationship between ferroptosis of tumor cells and T cells, it was discovered that IFNγ derived from CD8^+^ CTLs (Cytotoxic T lymphocytes) could downregulate SLC3A2 and SLC7A11 via JAK/STAT1 pathway (Wang W et al., 2019). Furtherly, IFNγ was found to increase the binding of the xCT transcription start site with STAT1 to inhibit its transcription. Thus, STAT1 deletion in tumor cells could reverse IFNγ-mediated reduction of xCT and ferroptosis (Wang W et al., 2019). Additionally, IFNγ could also activate ACSL4, thus promoting ferroptosis (Figure 7) (Zhang H. L. et al., 2022; Liao P et al., 2022).

### 5.2 Effects of macrophages on ferroptosis of tumor cells

In ovarian cancer, Ferroptosis-related genes (FRGs) that promote ferroptosis, such as FTH1, HIF1A, xCT and GPX4, are greatly correlated with cancer poor prognosis and tumor assoociated macophages (TAMs) infiltration. And in bladder cancer, ferroptosis-promoting FRGs that are involved with iron storage or uptake, including CISD1, CDGSH and FDFR3, have also been found correlated with an increased TAMs infiltration (Liang J et al., 2021; Wang S et al., 2021; Yan Y et al., 2021; You Y et al., 2021; Shao Y et al., 2021; Hu ZW et al., 2021). The ferroptosis repressor FTH1 and the driver SOCS1 were associated with M2 and M1 infiltration respectively in head and neck carcinoma (Hu ZW et al., 2021). Some lncRNAs correlated with ferroptosis were also found associated with TAMs infiltration in hepatocellular carcinoma (Liang J et al., 2021). All these results indicate that there exists tight correlations between macrophages or macrophage polarization with tumor ferroptosis.

M1 type macrophages can make tumors more sensitive to oxidative stress and activate tumor clearance immune responses within the tumor immune microenvironment (Pan Y et al., 2020). According to previous studies, there are at least three mechanisms for this effect: activating CD8^+^ T cells, releasing various cytokines, and directly dicharging peroxides such as H2O2 produced from respiratory burst processes (Emam M et al., 2021; Forman and Torres, 2002; Jakubczyk K et al., 2020; Davies LC et al., 2019). One type of cytokines which can be secreted by M1, IL-6, could cause iron acculmulation via promoting the producetion of hepcidin which can bind to transmembrane iron exporter via JAK/STAT3 pathway (Fraenkel, 2017; Zhang Z. et al., 2022). IL-6 can also downregulate xCT by activating extracellular regulatory protein kinase (ERK) signaling (Yang et al., 2020b). But in another study, it was found IL6 could promote tumor progression by inhibiting ferroptosis in the case of HNSCC (Li et al., 2022).

In contrast, M2 type macrophages are more likely to regulate ferroptosis in an indirect way by inhibiting the prompting function of CTLs on tumor cells ferroptosis. Through inhibting CXCL9, CXCL10 and CXCL12, M2 could inhibit the recruitment and activation of CTLs (Petty et al., 2019; Das S et al., 2020); M2 can also upregulate PD-L1 of neoplastic cells (Wen Z et al., 2018; Yun J et al., 2020), causing more T cell deaths. MXRA8, a newly discovered molecular matrix remodeling-associated protein, could reduce the intracellular Fe2+ level and lipid peroxidation, simultanously increasing M2 infiltration in glioma cells (Xu Z et al., 2022), further supporting the idea that M2 could inhibit ferroptosis of tumors. However, some cytokines that could be secreted by M2 in large amounts may enhance ferroptosis. For instance, TGF-β1 can promote the process of transporting electrons from NADPH to O2 o generate more ROS via activating (Carmona-Cuenca I et al., 2008). And TGF-β1 can activate Smad3 to downregulate xCT (Kim et al., 2020).

### 5.3 Effects of ferroptotic tumor cells on the immune system

Damage associated molecular patterns (DAMPs) are a set of molecular products that would interact with theri corresponding pattern recognition receptors (PRRs) distributed on monocyte surface. PRRs work via receiving these information conveyed through DAMPs, subsequenly generating corresponding reactions.

What’s noteworthy is that different DAMPs released by ferroptotic tumor cells may send totally different information. It could produce immunogenic cell death, leading to anti-tumor immunity (Galluzzi L et al., 2017); while in some other conditions, it promotes some responses supporting tumor growth (Tang D et al., 2012). For example, ferroptosis-associated lipid peroxides can enhance dendritic cells’ antigens recognition, phagocytosis and processing capacity, presenting those antigens towards CD8^+^ T lymphocytes to activate CTLs, thus enhancing antitumor immunity (Linkermann A et al., 2014; Tang R et al., 2020; Aaes TL et al., 2016). 1-steaoryl-2-15-HpETE-sn-glycero-3-phosphatidylethanolamine (SAPE-OOH), the oxidized lipid product released by ferroptotic cells, could interact with macrophages toll-like receptor 2 (TLR2), promoting M1 activation in ovarian cancer (Luo X et al., 2021). Ferroptosis-associated autophagy released High-mobility group box 1 (HMGB1) (Xue J et al., 2021) can not only elevate M1 polarization, but also enhance TNF-α secretion of TAMs in bladder cancer mediated by advanced glycosylation end product-specific rereceptor (AGER) (Wen Q et al., 2019). Conversely, pancreatic ductal adenocarcinoma (PDAC) cells that are ferroptotic could release protein KRAS-G12D in the form of exosomes, and it is further taken up by AGER on macrophages, promoting STAT3-dependent M2 polarization (Dai E et al., 2020). Another DAMP, 8-hydroxy-2′-deoxyguanosine (8-OHG), generated from oxidation-related DNA impair, was discovered to activate transmembrane protein and the downstream DNA sensor pathway, promoting M2 infiltration, thus favoring PDAC tumor progression. Cancer cells undergoing ferroptosis could induce phagocytosis of bone marrow-derived dendritic cells phagocytosis by specific DAMPs (ATP, HMGB1 and CRT) (Turubanova VD et al., 2019).

## 6 Tumor progression and ferroptosis

Loads of different gene expression modes and signals could change the level of susceptibility to ferroptosis of tumor cells, thus affecting tumorigenesis and their progression viability.

### 6.1 RAS

**FIGURE 8:**
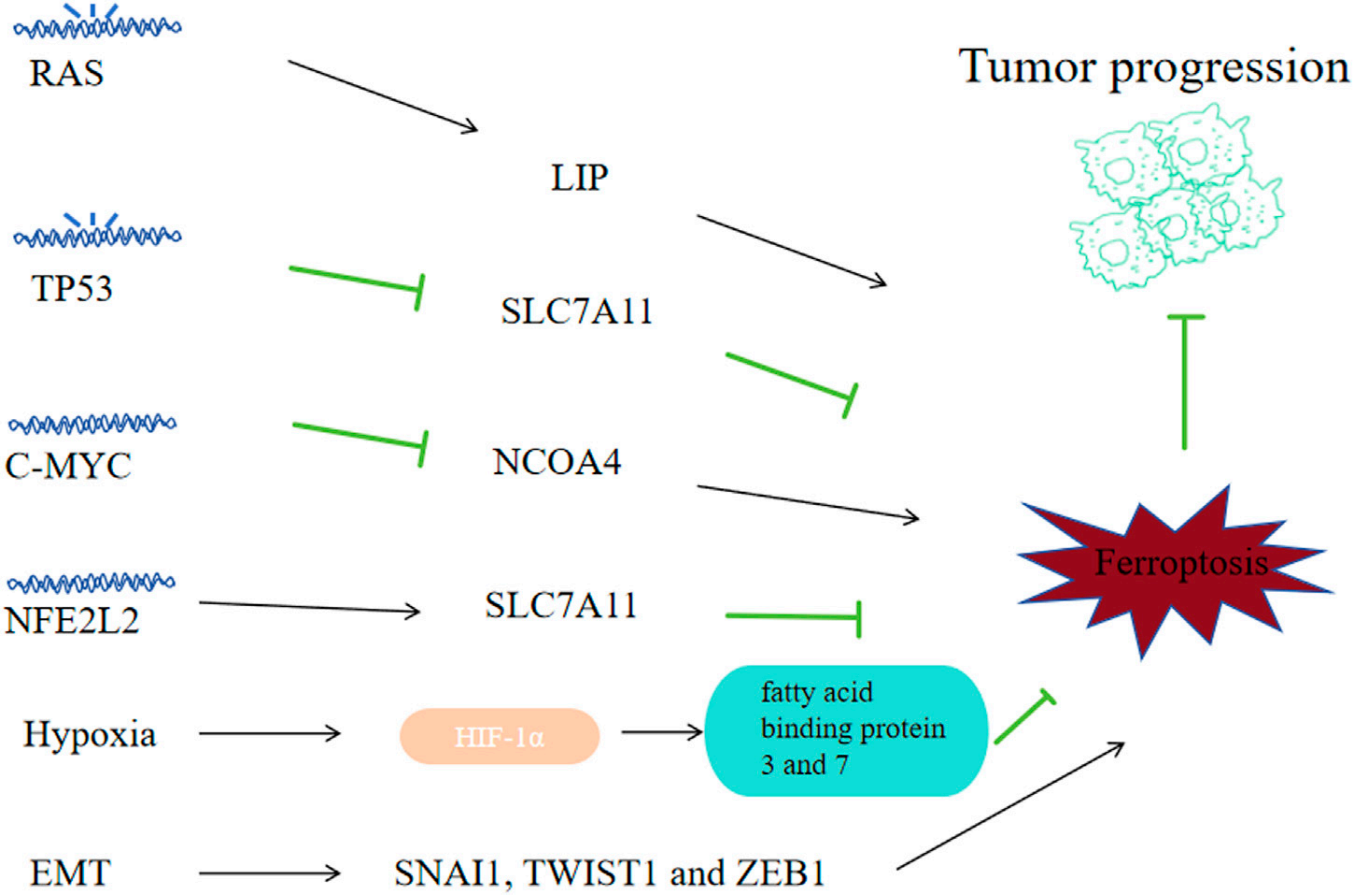
Correlation between ferroptosis and tumor progression. Some tumor oncogenes, suppressor genes and physiological phenomena-related signals may affect tumor cell sensitivity to ferroptosis, thus affecting tumor progression. Mutated RAS promotes ferroptosis by regulating iron metabolism genes; Mutated TP53 can promote ferroptosis by downregulating SLC7A11; C-MYC can inhibit ferroptosis by suppressing NCOA4 and inhibiting mitophagy; NFE2L2 can inhibit ferroptosis by upregulating SLC7A11; Hypoxia helps to accumulate HIF1α and upregulates fatty acid-binding proteins 3 and 7, inhibiting ferroptosis; High transcriptional levels of EMT-related SNAI1, TWIST1 and ZEB1 come with high sensitivity to ferroptosis.

The RAS family oncogenes, namely, KRAS, NRAS and HRAS, represent the most prevalently mutated genes across human cancers (Ryan and Corcoran, 2018). The study of erastin’s effects on 117 cancer cells revealed the mechanisms of Ferroptosis that is RAS dependent and RAS independent (Yang WS et al., 2014). Erastin and RSL3, the ferroptosis inducers, exhibit selective toxicity towards tumor cells that are engineered with RAS mutantion (Yang and Stockwell, 2008; Dolma S et al., 2003). And genetic and pharmacological blockade on RAS and its downstream molecules (ERK, MEK and BRAF) could reverse the anti-tumor effects of erastin and RSL3 (Yang and Stockwell, 2008; Yagoda N et al., 2007). The reason for RAS mutated cancer cells showing high susceptibility to ferroptosis may be that the mutant RAS signals could improve the level of LIP via regulating the iron metabolism related genes expression like FTL, TFRCFTH1 and FTH1 (Figure 8) (Yang and Stockwell, 2008). For example, RAS mutant lung adenocarcinoma cells exhibit sensitivity to ferroptosis triggered by xCT inhibitors (Hu K et al., 2020). Cells derived from NSCLC with EGFR upstream mutations exhbit sensitivity towards ferroptosis (Poursaitidis I et al., 2017). And preclinical investigations found that ectopic expression of RAS mutants HRASV12, KRASV12 and NRASV12 downregulated ferroptosis in RMS13 rhabdomyosarcoma-derived cells (Schott C et al., 2015). So ferroptosis might be a considerable potential managing method for cancer with RAS mutations. And for RAS mutant cancer, there must also exist some mechanisms for them to combat against ferropotic chanllenges during the process of their growth, and those mechanisms and ways to block them need further exploration.

### 6.2 TP53

As a tumor suppressor gene, 50% of human cancers are accompanied with biallelic mutation or elimination of TP53, causing loss of wild-type p53 viability and unbridled tumor advancement (Bykov et al., 2018). P53, as a transcription factor, can promote or inhibit related mRNA synthesis by binding to specific gene promoters. P53 can downregulate the SLC7A11 transcription level (Jiang L et al., 2015) to promote ferroptosis of tumor cells. MDM2 and MDMX, two p53-binding proteins, can enhance ferroptosis on tumor cells in a p53-dependent way (Venkatesh D et al., 2020). TP53 alterations (polymorphisms or variation) would regulate p53’s capacity to elevate cell ferroptosis and apoptosis. P53 3 KR (K161R, K117R and K162R) acetylation-deficient mutants are incapable of inducing apoptosis; however, they could initiate the ferroptosis of lung cancer cells (Jiang L et al., 2015). While other acetylation-defective mutants, p53 4KR (K98R and 3KR) and p53 P47S (a polymorphism located in the p53’s N-terminal transactivation domain), could not induce ferroptosis (Wang S J et al., 2016; Jennis M et al., 2016). Although P53 R273H, R175H cuoldn’t bind to DNA, it could still inhibit SLC7A11 via weakening other transcription factors’ activity (Liu D S et al., 2017), suggesting there exists complex transcription factor networks mudulating the expression of ferroptosis regulators. P53 can regulate ferroptosis by affecting metabolism related genes like GLS2, FDXR88 and STA1 in various settings (Zhang J et al., 2017; Gao et al., 2015; Ou Y et al., 2016). In NSCLC-derived H1299 cell line, ALOX15 and ALOX12 were associated with ferroptosis induced via p53 (Hayano M et al., 2016). P53 could suppress NOX-regulated lipid peroxidation by binding to dipeptidyl-peptidase DPP4 of human colorectal cancer cells and inducing the expression CDKN1A expression in fibrosarcoma cells (Deacon, 2018; Xie Y et al., 2017). Restore p53’s viablity could be an effective way to fight cancer.

### 6.3 MYC

MYC gene is a set of oncogenes identified relative early, including C-MYC, L-MYC and N-MYC. C-MYC amplification is highly correlated with tumorigenesis and prognosis, the amplification of N-myc is implications for prognostic determination of tumors, while the amplification of L-MYC does not imply predisposition and prognosis in different tumors. C-MYC is a regulatory gene highly corelated with tumor cell apoptosis, proliferation and differentiation, and its overexpression can enhance the malignant phenotype expression in a variety of tumors. C-MYC suppresses ferroptosis via the autophagy of ferritin mediated by NCOA4, thereby decreasing ROS and preventing mitophagy in ovarian cancer cells (Jin Y et al., 2022). And in hepatocellular carcinoma cells, C-MYC inhibited glutamine deprivation-induced ferroptosis via increasing GOT1 and NRF2 (Zhao Y et al., 2023). Inhibiting C-MYC’s effects might be a strategy to reverse tumor’s progression.

### 6.4 NFE2L2

As a major regulator on the oxidative stress signaling pathway, NFE2L2 has dual effects on tcancer progression: low activity of NFE2L2 promotes tumorigenesis, while high NFE2L2 activity promotes cancer progression and treatment-resistant (Rojo de la Vega M et al., 2018). In cancer cells, The NFE2L2 expression is regulated not only vai Keap1-mediated degradation of protein, but also via oncogenic signaling pathways like KRAS-BRAF-MYC (DeNicola et al., 2011). Early preclinical researches suggest that NFE2L2 signaling is a significant defense pathway to ferroptosis and contributes to the development of resistance to sorafenib treatment on HCC cells (Sun et al., 2016a). As a multifunctional scaffold protein, Sequestosome1 could bind to Keap1 and inhibits Keap1 from binding to NFE2L2 that is newly synthesized during tumor ferroptosis. NFE2L2 can suppress oxidative injuries caused by ferroptosis via transactivating many cytoprotective genes, such as ROS detoxification enzyme related genes: NQO1, GSTP1, SESN2, AKR1C3, AKR1C2, AKR1C1, TXDRD1; GSH metabolism related genes: SLC7A11, CHAC1, GCLM and iron metabolism related genes: FTH1, HMOX1, MT1G, SLC40A1 (Anandhan A et al., 2020). So clinically we need to decrease the NFE2L2 level to promote the ROS level of cancer cells.

### 6.5 Hypoxia

Hypoxia promotes tumorigenesis and resistance to treatment. Hypoxia inducible factor 1(HIF), the main factor in hypoxia regulation, includes a constitutively expressed β subunit (ARNT) and an oxygen-unstable α subunit (including HIF1α, HIF2α/EPAS1, HIF3α) (Keith B et al., 2011). Under normoxia, EPAS1 and HIF1α are hydroxylated by EGLN family of HIFs, and underwent proteasomal degradation via the recognition of E3 ubiquitin ligase VHL. Under hypoxia, activating the hydroxylase leads to the accumulation of HIF1α and EPAS1 with ARNT and the formation of a heterodimer, consequently initiating the transcription of genes correlated with hypoxia adaptation and survival. The elevated expression of EPAS1 and HIF1α, exists in multiple kinds of tumors and is associated with their unfavorable prognosis (Keith B et al., 2011). Inhibitibg HIF signaling with molecules like 2-methoxyestradiol, BAY87-2243, PX478 and PT2385 has been investigated in clinical trials as a strategy against tumor growth (Courtney K D et al., 2018). The EGLN protein is an sensor of oxygen and cysteine that is dependent on iron, catalyzing the HIF hydroxylation (Ivan and Kaelin, 2017). Iron chelators can enhance HIF stability by inhibiting EGLN activity (Cho EA et al., 2013). In HT-1080 carcinoma fibrosarcoma cells, through upregulating fatty acid binding proteins 3 and 7, HIF1α enhanced the uptake of fatty acid, increased storage cability of lipid and inhibited lipid peroxidation, thus suppressed ferroptosis (Yang et al., 2019a). However, in RCC-derived cells, EPAS1 could promote ferroptosis by increasing HILPDA expression, thereby increasing the production of PUFAs and subsequent lipid peroxidations (Zou Y et al., 2019).

### 6.6 EMT

Epithelial mesenchymal transition (EMT) contributes to tumor stem cells generation, causing metastatic spread and promoting drug resistance towards clinical treatments (Yang et al., 2020). Transcription factors like ZEB1, TWIST1 and NAI1, all of which could be potentially used for tumor therapy, regulate EMT-mediated tumor metastasis and drug resistance. Apart from inhibiting the therapeutic effects of antitumor therapeutic approaches, EMT signalings can enhance ferroptosis (van Staalduinen J et al., 2018). High mesenchymal status and high sensitivity to ferroptosis are closely associated in human cancer cell lines and organoids (Viswanathan VS et al., 2017). High transcriptional level of ZEB1 is confirmed associated with high sensitivity to ferroptosis, part of which comes down to its capacity of elevating the expression of a main factor of lipid metabolism regulation in the liver, PPARγ(Viswanathan et al., 2017). Protein LYRIC/metadherin, which positively regulate EMT, promotes ferroptosis via inhibiting SLC3A2 and GPX4 (Bi J et al., 2019). CD44-dependent increase in iron endophagy would enhance iron-dependent methylase activity and EMT signalings related genes expression, which will in turn increase the vulnerability of breast cancer towards ferroptosis (Müller et al., 2020). The first step of EMT is to disrupt the contact between the epithelial cells. Cell contacts mediated by cadherin1 can inhibit ferroptosis (Wenz C et al., 2019; Wu et al., 2019; Yang M. et al., 2019). Other enhancers of cell adhesion, like integrin subunits β4 and α6, can inhibit ferroptosis of breast cancer *in vitro* (Brown CW et al., 2017). While ZEB1, TWIST1 and SNAI1 can restore the sensitivity towards ferroptosis (Wu et al., 2019). How to utilize the reltaionship of hight EMT activity and high sensitivity to ferroptosis is a major question since drug-resistant tumor cells show signs of EMT.

## 7 Feroptosis and tumor treatment

One of the characteristics of tumors is that they can resist or escape from apoptosis, which is unfavorable to tumor management. Good news is, these apoptosis-resistant tumor cells have a higher level of mesenchymal cell gene activity and are inactive at some essential genes to resist oxidative stress. Besides, these apoptosis-resistent cancer cells are sensitive to therapeutic regimens that target GPX4, implying the exclusive value of ferroptosis in tumor therapy-related studies. Furthermore, this hyper-mesenchymal cell status that is also resistant to treatments relies on the lipid peroxidase pattern, and is characterized by promoted polyunsaturated lipid synthesis and enhanced lipid peroxidase activity, and they are drug targetable (Viswanathan et al., 2017). In the other hand, cancer at different stages may exhibit different sensitivity to ferroptosis. For example, melanoma could be classified into four subcategories, and each has a distinct susceptibilty degree for ferroptosis, thus targeting the plasticity of differentiation to improve the outcome may offer a new prospect for cancer immunotherapy efficacy (Tsoi J et al., 2018).

### 7.1 Ferroptosis and tumor immunotherapy

Immune checkpoint inhibitors (ICI)-related immunotherapies function mainly by activating cytotoxic T cells (CTLs) on their anti-tumor effects, and the now-approved ICI can target CTLA-4, PD-L1 and PD-1. In patients of melanoma, reduced SLC3A2 expression is associated with a consistently high efficacy of ICI (Wang et al., 2019). Yet, Due to tumor microenvironment complexity and inadequate activation of immune system, ICI-related immunotherapies still has limited the rate of systemic anti-tumor response within a multitude of cancer types (Dong MB et al., 2019; Jiang Q et al., 2020). Interferons have dual functions of activating and suppressing immune responses, and the transmission of interferon signaling in immune cells and caner cells will confront each other. Obstruction of PD-1 inhibitors in melanoma, breast and colorectal cancer cells would prevent interferon signaling in tumor cells, enhancing the CD8^+^ T cells abundance, thus activating the immune system to combat tumor cells (Benci JL et al., 2019). What connects ferroptosis and ICI more closely is that PD-L1 antibodies could enhance ferroptosis on tumor cells, and liproxstatin1, the ferroptosis inhibitor, could downregulate the anti-tumor effects of these reagents (Wang et al., 2019). Ferroptosis inducers (erastin, cyst(e)inase and RSL3) and PD-L1 together can suppress tumor progress *in vivo* (Wang et al., 2019). In tumor tissue, the selective enrichment of nanoparticles that contain RSL3 could induce immunogenic death of the tumor cells, initiating anti-tumor immune response, simultanously inducing IFN-γ secretion by CTLs. Moreover, RSL-3 and IFN-γ can prevent the lipid peroxides repair pathway, enhancing the lipid peroxides buildup in the tumor cells. After immune checkpoint treatment, the CTLs infiltration in the tumor tissues can increase 4-fold, which productively curbed the tumor growth and metastasis (Song et al., 2021).

Except T cells, it is also a potential strategy to reporlarize TAMs for tumor immune therapies. There are many ways to repolarizes M2 type tumor TAMs to the M1 type, enhancing anti-tumor effects (Sindrilaru A et al., 2011), including microparticles released from tumor cells, sulfadiazine-loaded magnetic nanoparticles (Fe3O4-SAS@PLT), radiotherapy, TGF-β inhibitor, PD-1 antibody and the combining therapies of them (Jiang Q et al., 2020; Wan C et al., 2020).

### 7.2 Radiation therapy and ferroptosis

Some research studying the correlation between radiation and ferroptosis may shed some light on improving the ferroptosis-related tumor treatments. For example, one study found that radiation therapy can lead to KEAP1 gene mutations, upregulating SLC7A11 expression, thereby generating cellular resistance to radiotherapy and ferroptosis (Lei G et al., 2020). Suppression of GPX4 or SLC7A11 by FINs could greatly promote radiotherapy efficacy, reversing radiotherapy resistance (Lei G et al., 2020). While another study showed that radiotherapy activated telangiectasia mutant gene ATM, suppresses SLC7A11 expression, limits tumor cysteine uptake, reduces glutathione, increases lipid peroxidation levels, regulating ferroptosis of tumor cells in an opposite direction (Lang X et al., 2019). Radiotherapy can interact with effector T cells via ferroptosis to enhance tumor clearance. Immunotherapy activated CD8^+^ T cells could promote ferroptosis via IFNγ, and IFNγ could directly make tumor cells more sensitive to radiation therapy (Lang X et al., 2019).

### 7.3 Reagents or drugs associated with ferroptosis in tumor cells

Cancer tissues develop resistance to some anti-cancer drugs such as trametinib, lapatinib, erlotinib, dabbafenib and vimerafenib, to which ferroptosis may be an effective response strategy (Tsoi J et al., 2018; Hangauer MJ et al., 2017). Many tumor cells that are drug resistant show signs of EMT (elevated mesenchymal markers, downregulated epithelial markers), so they are more sensitive to ferroptosis. Ferroptosis inducers can also synergize with some conventional drugs like cisplatin to inhibit the progression in a mouse head and neck cancer model (Roh J L et al., 2016). Here, we will introduce several major drugs or agents that could manage tumor growth via inducing ferroptosis.

#### 7.3.1 Erastin

Erastin is selective toxic to RAS mutant cancer cells. Erastin can induce ferroptosis via several ways: binding to the subunits of Xc-to inhibit cystine transfer, or targeting VDAC, ACSL4 and p53 (Dixon et al., 2012). Although it can effectively induce ferroptosis, its poor water stability and solubility in physiological environment makes it not suitable for clinical use (Dixon et al., 2012). While some research suggested that some combination therapy with erastin holds potential in application. For example, erastin is capable of enhancing the sensitivity of lung cancer cells to the drug cisplatin, and could improve lung cancer patients’ survival rate, enhancing its therapeutic efficacy when treated with epidermal growth factor receptor tyrosine kinase inhibitors (EGFR-TKIs).

#### 7.3.2 Sorafenib

Approved by the FDA, sorafenib was a multi-tyrosine kinase inhibitor used for unresectable HCC patients, advanced renal cell carcinoma and differentiated thyroid cancer. Sorafenib was evaluated in monotherapy and combination therapies with conventional cytotoxic drugs in several malignancies. As a multikinase inhibitor, sorafenib inactivates the kinase required for Xc-. Sorafenib could suppress many cell surface kinases (PDGFRB, VEGFR1-3, RET, FLT3 and KIT) and intracellular kinases (wild-type, RAF and mutant BRAF). Several researches have shown that sorafenib can induce autophagy and apoptosis via targeting these kinases in prostate cancer and liver cancer (Ullén et al., 2010; Garten A et al., 2019). Sorafenib was initially believed to inactivate many of the oncogenic kinases to induce ferroptosis of HCC. However, several researches involving lung, kidney, liver and pancreas cancer-derived cells indicate that it induces ferroptosis primarily by inhibiting Xc-(Sun et al., 2016b; Dixon et al., 2014). Even with a low concentration, sorafenib combining aspirin resulted in GSH depletion, Xc-inhibition, and ROS buildup in cancer cells, simultanously improving cisplatin cytotoxicity to drug-resistant cells via inhibiting DNA damage. Preclinical and clinical researches confirmed that the target genes of NFE2L2-MT1G are the biomarkers accountable for sorafenib resistance (Sun et al., 2016a; Houessinon A et al., 2016), and found that via promoting ferroptosis of human HCC cells, MT1G knockout restores the efficacy of sorafenib in combating tumor. The increased levels of ACSL4 were accompanied by the high sensitivity of HCC cells towards sorafenib, suggesting that rosiglitazone, the ACSL4 inhibitor, rosiglitazone may block the anti-tumor effects of sorafenib (Feng J et al., 2021; Kagan VE et al., 2017; Doll S et al., 2017). However, it stays unknown that how much the extent to which apoptosis or ferroptosis promote the anti-tumor effects of sorafenib in clinical models.

#### 7.3.3 Sulfasalazine

An oral anti-inflammatory drug, sulfasalazine was used for inflammatory bowel diseases (including ulcerative colitis and Crohn’s disease) and rheumatoid arthritis (Fleig WE et al., 1988). The mechanism of sulfasalazine and its metabolites (5-aminosalicylate and sulfonalazine) are still in the study, and it may be related to lymphocyte inhibition (e.g., inhibition of B cell-mediated antibody production or induction of T cell death) and leukocyte regulation like inhibiting leukocyte recruitment, subsequent prostaglandin biosynthesis and cytokine production (Rachmilewitz D et al., 1978; Smedegård and Björk, 1995). Apart from sulfasalazine’s anti-inflammatory capacity, it could also suppress the progression of lymphoma and other cancer via suppressing Xc-in preclinical models (Gout PW et al., 2001; Dixon et al., 2012). Sulfasalazine can effectively deplete GSH in colorectal cancer cells, enhance the anti-tumor activity of cisplatin, cause a great buildup of ROS, and suppress the progression of colorectal cancer (Ma MZ et al., 2015). Sulfasalazine causes ferroptosis of glioma cells through inhibiting cysteine uptake. However, in preclinical studies, it is difficult to determine whether the anti-tumor capacity of sulfasalazine is dependent on Xc-inhibition or its anti-inflammatory effect, as the structural basis for its pharmacological effects is unclear (Gadangi P et al., 1996; Sehm T et al., 2016). Glioblastoma is a kind of brain tumor that is very aggressive, and it often causes recurrence and death even after radiotherapy and surgery. In glioma cells, sulfasalazine promotes ferroptosis via inhibiting cysteine uptake regulated by Xc-.

## 8 Conclusion

Ferroptosis has shown great values in helping comprehend the mechanisms of tuomr progression and it has irreplaceable potentials in tumor management. Recent studies have greatly improved our understanding of ferroptosis. But studies on ferroptosis are still in enormous need because its occurrence and regulation mechanisms are not fully discovered. And we need to explore more ferroptosis inducers and to develop combination therapies for ferroptosis with exosomes, nanodrugs and tumor immunotherapy to treat cancers.
